# The effect of hypoxia on myogenic differentiation and multipotency of the skeletal muscle-derived stem cells in mice

**DOI:** 10.1186/s13287-022-02730-5

**Published:** 2022-02-05

**Authors:** Mohamed I. Elashry, Mebrie Kinde, Michele C. Klymiuk, Asmaa Eldaey, Sabine Wenisch, Stefan Arnhold

**Affiliations:** 1 Institute of Veterinary Anatomy, Histology and Embryology, Justus-Liebig-University of Giessen, Frankfurter Str. 98, 35392 Giessen, Germany; 2 Clinic of Small Animals, c/o Institute of Veterinary Anatomy, Histology and Embryology, Justus Liebig University of Giessen, 35392 Giessen, Germany; 3 Anatomy and Embryology Department, Faculty of Veterinary Medicine, University of Mansoura, Mansoura, 35516 Egypt

**Keywords:** Muscle Stem cells, Proliferation, Differentiation, Multipotency, Hypoxia

## Abstract

**Background:**

Skeletal muscle-derived stem cells (SC) have become a promising approach for investigating myogenic differentiation and optimizing tissue regeneration. Muscle regeneration is performed by SC, a self-renewal cell population underlying the basal lamina of muscle fibers. Here, we examined the impact of hypoxia condition on the regenerative capacity of SC either in their native microenvironment or via isolation in a monolayer culture using ectopic differentiation inductions. Furthermore, the effect of low oxygen tension on myogenic differentiation protocols of the myoblasts cell line C2C12 was examined.

**Methods:**

Hind limb muscles of wild type mice were processed for both SC/fiber isolation and myoblast extraction using magnetic beads. SC were induced for myogenic, adipogenic and osteogenic commitments under normoxic (21% O_2_) and hypoxic (3% O_2_) conditions. SC proliferation and differentiation were evaluated using histological staining, immunohistochemistry, morphometric analysis and RT-qPCR. The data were statistically analyzed using ANOVA.

**Results:**

The data revealed enhanced SC proliferation and motility following differentiation induction after 48 h under hypoxia. Following myogenic induction, the number of undifferentiated cells positive for Pax7 were increased at 72 h under hypoxia. Hypoxia upregulated *MyoD* and downregulated *Myogenin* expression at day-7 post-myogenic induction. Hypoxia promoted both SC adipogenesis and osteogenesis under respective induction as shown by using Oil Red O and Alizarin Red S staining. The expression of adipogenic markers; *peroxisome proliferator activated receptor gamma* (*PPARγ*) and *fatty acid-binding protein 4* (*FABP4*) were upregulated under hypoxia up to day 14 compared to normoxic condition. Enhanced osteogenic differentiation was detected under hypoxic condition via upregulation of *osteocalcin* and *osteopontin* expression up to day 14 as well as, increased calcium deposition at day 21. Hypoxia exposure increases the number of adipocytes and the size of fat vacuoles per adipocyte compared to normoxic culture. Combining the differentiation medium with dexamethasone under hypoxia improves the efficiency of the myogenic differentiation protocol of C2C12 by increasing the length of the myotubes.

**Conclusions:**

Hypoxia exposure increases cell resources for clinical applications and promotes SC multipotency and thus beneficial for tissue regeneration.

**Supplementary Information:**

The online version contains supplementary material available at 10.1186/s13287-022-02730-5.

## Introduction

Muscle-derived stem cells (SC) have received considerable attention to investigate myogenesis, tissue regeneration and stem cell therapy. SC are known as satellite cells, a self-renewing cell population beneath the basal lamina of muscle fibers [1]. SC are the main cell population responsible for muscle regeneration. Several evidence have reported the negative effect of muscle degeneration on the quality of life as shown in muscular dystrophy and sarcopenia; an age-related loss of muscle mass [2, 3]. It was documented that 20–25 per 100,000 persons suffer from muscular dystrophies worldwide [4] and 25% and 40% of individuals older than 70 and 80 are more susceptible to sarcopenia respectively [3]. So far, muscle degenerative diseases have no convincing cure [5–7]. The current approaches are more likely to limit progression of muscle loss [3]. Stem cells offer a great potential for the treatment of incurable diseases which cannot be handled by conventional medicine [8–11]. Additionally, tracking SC performance in the course of myogenic differentiation either as single cells cultivated in a monolayer or when the cells remain in contact with the muscle fiber do not only facilitate the understanding of the regenerative process but also optimize the microenvironment in vitro to mimic in vivo conditions [12–14].

SC are quiescent under normal conditions [1, 15–17]. Up on muscle injury, the cells enter cyclic proliferation with the expression of MyoD to form sufficient myoblasts that either differentiate to restore the myonuclear loss in the preexisting myofibers or form new myofibers [14–19]. Cell proliferation and differentiation are orchestrated by myogenic regulatory elements including Pax7, MyoD, Myf5, Myogenin and MRF4 expression [20]. Quiescent SC are positive for Pax7, an essential factor for their maintenance and self-renewal [21, 22]. Committed myoblasts exit from the cell cycle by downregulation of Pax7, and upregulation of Myogenin and MRF4 expression [20, 23]. Meanwhile, MyoD regulates the transition from undifferentiated myogenic precursors to myoblasts, while Myogenin is involved in myoblasts fusion to form multinucleated myofibers [24]. At the end of myogenesis, Myogenin initiates the expression of the contractile elements particularly myosin heavy chain (MHC) proteins [23]. On the other hand, SC showed commitments into adipogenic and osteogenic fate using specific inducers [25].

For effective clinical application, an in vitro expansion and characterization of a suitable SC population is required [2]. However, myoblasts expansion in culture is very much influenced by factors in the culture condition [26, 27]. Oxygen is one of the basic components of the microenvironment that plays an important role for cellular metabolism, growth and differentiation [28, 29]. A large body of evidence have shown that hypoxia (HX) enhanced proliferation and differentiation in primary isolated myoblasts in mice [30], rat [31] and bovine [29]. Although the physiological oxygen concentration within the adult skeletal muscle tissue is almost 3.8%, most of the in vitro studies were carried out under 21% oxygen which is by far higher than the endogenous requirement in the cellular microenvironment [32]. An abundance of evidence indicate that alteration of the oxygen levels can modulate the regenerative capacity of stem cells in vitro [29, 33]. Furthermore, the composition of the culture components can also influence the differentiation potential of myoblasts [22]. Most of the previous studies have been generated on isolated SC in a monolayer culture, whereas SC have been removed from their natural niche. Furthermore, a detailed report investigating the effect of HX on the myogenic differentiation (MD) and multipotency of SC are not fully elucidated. Thus, it is essential to dissect the effect of HX on SC either in their native niche mimicking the physiological microenvironment or even after isolating SC as monolayer culture condition. Furthermore, it is important to examine the MD protocol under optimized O_2_ condition in vitro before further clinical application. Thus, the present study aimed to evaluate the effects of HX on the viability, proliferation, and multipotency of SC as well as, to assess the efficiency of the MD protocols under HX compared with normoxia (NX). The data revealed enhanced cell viability, cell motility along the muscle fiber and proliferation capacity under 3% HX that facilitate in vitro reproducibility of SC before clinical applications. We show impaired MD under HX due to failure of cell withdrawal from cyclic division and terminal differentiation. We provide evidence of promoted adipogenesis and osteogenesis under HX suggesting that targeting HX might be a candidate for improving bone regeneration. We found that adding dexamethasone to the myogenic protocol overcomes the negative effect of HX on myogensis, and it could be an approach to improve muscle regeneration. Taken together, our data reveal a positive effect of HX on SC proliferation, motility and multipotency. Thus, optimized HX exposure could be beneficial not only to improve cell resources before transplantation but also to promote multipotential differentiation of muscle stem cells to fat and bone formation.

## Materials and methods

### Cell culture

#### Isolation of SC with or without intact muscle fiber

Single muscle fibers were generated from *M. extensor digitorum longus* of mice (n = 6) as previously reported [34, 35]. All husbandry and experimental procedures were approved by the institutional ethics committee and the Regierungspräsidium Gießen (GI 20/10 Nr. 105/2014). Briefly, muscles were isolated with both intact tendons then were digested in 0.2% collagenase IV (Biochrome, Germany) in Dulbecco’s modified Eagle’s medium (DMEM, Gibco, Germany) with 1% penicillin / streptomycin (P/S, Gibco, Germany) at 37 °C for 90 min. SC were extracted from hind limb muscles including *M. tibialis anterior, M. gastrocnemius and M. soleus*. Muscles were washed in cold PBS containing 1% P/S three times for 5 min each then were digested in 0.1% collagenase IV in DMEM supplemented with 1% P/S at 37 °C for 45 min with shaking. The homogenates were centrifuged at 2000 × g for 5 min. Supernatants were discarded and pellets were suspended in 2% fetal calf serum (FCS, Biocell) and then were filtered in 70-µm cell strain. Filtrates were centrifuged at 2000 × g for 10 min. The cell pellets were suspended in fresh 2% FCS/DMEM. Selection of SC based on surface markers including CD44, CD90 and α7-integrin expression was performed using magnetic beads as previously reported from our group [36, 37]. Briefly, 10 µL of goat anti mouse IgG magnetic bead solution was incubated with 5–10 µg of mouse CD90, CD44 (1:50, DSHB) and α7 integrin (1:50, Santa Cruz) primary antibodies in 5% FCS/PBS with at 4 °C for 1 h. After three successive washing in PBS, 1 × 10^7^ cells were incubated with the antibodies beads mixture at 4 °C for 30 min. Selection of beads-coated cells was carried out using a magnetic field. Harvested cells were washed in PBS and then were characterized using immunohistochemistry and PCR.

#### C2C12 myoblasts

The Mouse myoblasts C2C12 cell line is a subclone of C2 myoblasts originally isolated from thigh muscles of eight weeks-old mice as previously reported [38, 39]. The cells were purchased from American Type Culture Collection (ATCC, Germany). Cells were thawed at 37 °C in a water bath and then were expanded in growth medium (GM) composed of DMEM containing 4.5 g/mL D-glucose, L-glutamine and pyruvate (Gibco, Germany) supplemented with 10% FCS and 1% P/S at 37 °C under standard culture condition. Upon 60% confluency, cells were detached from the culture flasks using TrypLE™ express (Gibco, Denmark) and were counted using a hemocytometer under inverted phase contrast microscope. The cells were either seeded in plates or passaged based on the experimental setup.

### Assessment of cell viability

MTT (3-(4, 5-Dimethylthiazol-2-yl)-2, 5-Dimethyltetrazolium Bromide) assay measures the metabolic activity of cells that indicates the number of viable cells. C2C12 mouse myoblasts were seeded in 24-well culture plates at the rate of 1 × 10^4^ cells/ well in GM. Cells were incubated under both NX (21% O_2_) and HX (3% O_2_) for 1, 4, and 7 days. Cells were provided with fresh medium every two days. Furthermore, cells were incubated with 0.5 mg/mL MTT (Sigma-Aldrich, Germany) at 37 °C for 2 h. MTT solution was discarded, and the cells were incubated with 200 μL/well of dimethyl sulfoxide for 10 min with shaking at room temperature (RT). Lysates were pipetted in triplicates into a 96-well microplates. The absorbance was measured at 570 nm using a microplate reader (Tecan, Switzerland).

### Sulforhodamine B assay

Sulforhodamine B (SRB) assay semi-quantifies the amount of cellular protein contents which is considered as a proportional indicator to the cells number [40]. Briefly, 1 × 10^4^ cells/well were seeded in 24-well plates in GM at 37 °C and 5% CO_2_ under NX and HX conditions. Cells were provided with fresh medium every two day. Cells were fixed with 4% paraformaldehyde (PFA, Roth, Germany) for 10 min at RT at days 1, 4, and 7. Cells were incubated with 0.4% SRB sodium salt (Sigma-Aldrich, Germany) diluted in 1% acetic acid (Merck, Germany) for 10 min at RT. Non-buffered Tris solution (10 mM, pH 10, Sigma-Aldrich, Germany) was added into each well and was incubated for 30 min at RT to dissolve the bounded SRB. The solution was transferred into a 96-well plate, and the absorbance was measured at 565 nm using the microplate reader (Tecan, Switzerland).

### Colony forming unit assay

The colony forming unit assay was performed to evaluate the proliferative capacities of C2C12 myoblasts following HX exposure. C2C12 myoblasts were seeded in 7 mL of GM per T-25 flasks at seeding densities of 1 × 10^2^, 5 × 10^2^, 1 × 10^3^ and 5 × 10^3^ cell per flask. Cells were incubated under both NX and HX culture conditions at 37 °C for seven days with medium change every two days. Cells were fixed with 4% PFA for 10 min at RT then were stained with 5 mg/mL crystal violet (Roth, Germany) diluted in 2% ethanol (Roth, Germany) for 8 min. Stained cells were washed twice for 10 min using distilled water and were left to dry overnight at RT. The number of colonies were counted via the inverted light microscope equipped with digital camera; however, the size of colonies (μm^2^) was measured using ImageJ software.

### Induction of multipotential differentiation

SC were seeded either with intact muscle fiber (15–20 fibers/well) or isolated myoblasts 1 × 10^4^ cells/well in triplicates (n = 6) in 24-well culture plates. In the latter, the monolayer was allowed to expand in GM for 48 h before induction. Cells were divided into two experimental groups cultivated under NX and HX conditions using oxygen tension controlled chamber (Labortect Incubator C16, Göttingen, Germany). The differentiation was performed using MD medium consisting of 4.5 g/L glucose DMEM supplemented with 2% horse serum (Millipore, Darmstadt, Germany), 2.5 ng/mL human Fibroblast Growth Factor (Invitrogen) and 1% sodium pyruvate (Sigma-Aldrich, Germany) up to 14 days. Multipotential differentiation was carried out using either adipogenic differentiation medium (AD) composed of 4.5 mg/mL glucose DMEM supplemented with 5% FCS, 1 µM dexamethasone (Sigma-Aldrich), 5 µg/mL Insulin-transferrin-selenium (ITS, Sigma-Aldrich, Germany) and 5 µM Rosiglitazone (Sigma-Aldrich, Germany) or osteogenic differentiation medium (OD) contained 1 mg/mL glucose DMEM supplemented with 5% FCS, 0.1 µM dexamethasone, 250 µM ascorbic acid (Sigma-Aldrich) and 10 mM β-glycerophosphate (Fluka, Germany). Non-induced cells were kept in basal medium (BM) containing 5% FCS in DMEM and 1% P/S were considered controls. SC/fibers were allowed to differentiate up to 72 h (Additional file 1: Video S1), whereas extracted myoblasts for monolayer culture were differentiated in AD and OD medium up to 21 days.

### Myogenic differentiation protocols

To assess the effect of HX on the efficiency of the MD protocol, C2C12 differentiation was performed using modified MD protocols. Briefly, C2C12 cells were seeded in 24-well culture plates at a rate of 1 × 10^4^ cells per well in GM and were incubated at 37 °C in 5% CO_2_ and 21% oxygen concentration. Up to 60–70% confluency, MD was induced using MD medium supplemented with either of the following elements; 1 mM dexamethasone, 5 ng/mL transforming growth factor beta (TGF β) (Sigma-Aldrich, Germany), 1 mg/mL insulin, 0.57 mg/mL transferrin and 0.5 μg/mL sodium selenite (ITS). The cells were allowed to differentiate under NX and HX conditions for evaluation at day-4 and day-14 post-induction.

### Immunohistochemistry

SC/fibers cultivated in MD, AD and OD medium under both NX and HX conditions were fixed at 48 h and 72 h in 4% PFA for 10 min at RT. The cells were washed twice in PBS for 5 min each and then were permeabilized in a buffer composed of 20 mM Hepes, 300 mM sucrose, 50 mM NaCl, 3 mM MgCl_2_ and 0.5% Triton™ X-100 (pH 7, Calbiochem GmbH, Germany) for 15 min. The cells were blocked in a buffer containing 5% FCS diluted in PBS, 0.05% Triton™ X-100 for 30 min. The cells were incubated with mouse anti-Pax7 (1:30, DSHB, USA), MyoD (1:100, BD Biosciences) and Myogenin (1:30, DSHB, USA) primary antibodies at 4 °C overnight. The cells were washed for 1 h with the blocking buffer then were incubated with goat anti-mouse IgG Cy3 and goat anti-mouse fluorescent isothiocyanate (FITC) (1:200, Dianova, Germany) secondary antibodies in dark at RT for 1 h. SC/fiber were mounted on slides using DABCO Mowiol (Roth, Germany) and were examined using Axio-imager fluorescent microscope operated with Axiovision software (Zeiss, Germany). The number of Pax7, MyoD and Myogenin positive cells/fiber and number of SC clusters/fiber were manually counted (n = 15–20 fibers per experimental condition). Immunofluorescence for C2C12 to detect the myogenic relative markers and the contractile proteins was carried out. Briefly, C2C12 cells were cultivated in MD medium at a rate of 1 × 10^4^ cells/well on sterile glass coverslips in 24-well culture plates for 1, 4 and 7 days under both NX and HX conditions. Immunofluorescence staining for Pax7 and MyoD was performed on day 1 and 4, while staining for Myogenin was carried out on day 7 after differentiation. The staining of MHC types was examined at day 14 following MD using mouse anti-MHC type I, MHC type IIa and MHC type IIb (1:30, DSHB) primary antibodies diluted in the blocking buffer. Nuclei were visualized using 4, 6-diamidino-2-phenylindole dihydrochloride (DAPI, ThermoFisher).

### Phalloidin staining

To evaluate the effects of HX on the morphometric parameters of C2C12 cells, 1 × 10^4^ cells per well were seeded in GM on coverslips. The plates were cultivated under both experimental conditions up to day-14 post-MD. The cells were fixed with 4% PFA for 10 min at RT then were washed in PBS twice for 10 min. the cells were permeabilized in 0.1% tween (Roth, Germany) for 5 min. After washing with PBS, the cells were incubated with 0.5 mg/mL fluorescein isothiocyanate conjugated phalloidin (Sigma-Aldrich, Germany diluted 1:30 in PBS) in dark for 30 min. Cell nuclei were counterstained with DAPI for 5 min. The cells were mounted with DABCO Mowiol and then were photographed under fluorescent microscope. Morphometric analysis of the myotubes indicating MD was conducted on five randomly chosen microscopic fields per experimental group (n = 6). The data parameters including number, length and size of the myotubes were measured using ImageJ software.

### Alizarin Red S staining (ARS)

The cells induced to osteogenic lineage under NX and HX conditions up to 21 days were fixed in 4% PFA in PBS for 10 min then, were washed in distilled water for 5 min, were incubated with 1% ARS (pH 4.2) at RT for 10 min and were washed three times with distilled water for 5 min. The mineralized matrix were examined and were photographed using the light microscope equipped with camera and LAS V4. 4 software (Leica, Germany). Semi-quantitative evaluation of ARS at days 14 and 21 was carried out. Briefly, the stained plates were washed twice with distilled water and then were incubated with 2 mL of 10% Cetyl Pyridinium Chloride (Roth Germany) for 1 h to elute the staining. From each experimental group (n = 6), a volume of 200 μL was transferred to a 96-well plate in triplicates. The absorbance was detected at 562 nm using a microplate reader (Tecan, Germany).

### Oil Red O staining (ORO)

Following adipogenic induction under NX and HX conditions up to 21 days, the cells were fixed in 4% PFA for 10 min, were washed in PBS and were incubated with ORO staining (Sigma-Aldrich) diluted in distilled water for 30 min. ORO stained cells were examined and were photographed using light microscope provided with a digital camera and the LAS V4.4 operating software (Leica, Germany). Semi-quantitative analysis for adipogenic capacity of the cells was performed. Briefly, ORO stained cells were incubated with 100% isopropanol for 30 min. A volume of 200 µL from each experimental group was pipetted into a 96-well plate and the absorbance was measured at 492 nm in triplicates using the microplate reader. For both experimental groups, the number of adipocytes (n), the number of fat vacuoles per adipocyte (n) and the size of the fat vacuoles (µm^2^) per adipocyte at days 14 and 21 were measured. Five microscopic images representing the whole well plate were analyzed using ImageJ software.

### RT-qPCR

Cells induced to MD, AD and OD under both NX and HX conditions were evaluated up to 21 days post-induction. Non-induced cells kept in BM were processed in parallel, and served as negative controls. Almost 1 µg RNA probes were extracted from all experimental groups using RNA purification kit (Sigma-Aldrich, Germany). RNA samples were treated with DNAse I (Roche, Germany) and then were reverse-transcribed into cDNA by using a Multiscribe™ Reverse Transcriptase (Thermo Fisher Scientific). The cyclic conditions including; 21 °C for 8 min, 42 °C for 15 min, 99 °C for 5 min, and 5 °C for 5 min, followed by cooling step to 4 °C were applied. The harvested cDNA was incubated with Biotherm Taq Polymerase, ultrapure water, buffer, dNTP, and the relative forward and reverse primers. The primers used in the current study are random hexamers (Table 1, Microsynth, Germany). PCR was carried out for 35 cycles as follows; 5 min at 95 °C, 30 s at 94 °C, 30 s at 60 °C, 30 s at 72 °C, and 1 min at 72 °C. Quantification of MD (*MyoD* and *Myogenin*), AD (*peroxisome proliferator activated receptor gamma, PPARγ* and *fatty acid-binding protein 4*, *FABP4*), OD (*osteocalcin*, *OC* and *osteopontin*, *OP*) and *hypoxia inducible factor 1 alpha* (*HIF1α*) markers expression was measured in triplicate via RT-qPCR using the GoTaq qPCR Mix (Promega, Germany). A thermal cycler was operated for 2 min at 95 °C, 15 s at 95 °C, 30 s at 60 °C, 5 s at 60 °C, and 5 s at 95 °C for 40 cycles using Bio-Rad CFX Manager 2.1 software (Bio-Rad GmbH, Germany). *18S1* was served as an endogenous reference, and the relative expression was assessed using the 2- ^∆∆CT^ method [41].

### Statistical analysis

The data were collected from three independent experiments (n = 6). To analyze the effect of differentiation medium (MD, AD and OD *vs.* BM) on the number of Pax7, MyoD, Myogenin positive cells and clusters per muscle fiber at 48 and 72 h under both NX and HX conditions, a two-way ANOVA was performed. Evaluation of the viability of isolated SC following ectopic differentiation (MD, AD and OD *vs.* BM) after two weeks under NX and HX conditions, a one-way ANOVA was carried out. To analyze the semi-quantification measurements of OD and AD staining at days 14 and 21 under both experimental conditions, a two-way ANOVA was used. To evaluate the effect of HX on the number of adipocytes as well as the number and size of fat vacuoles per adipocyte following AD at day 14 compared to NX culture, a two-tailed t test was performed. Analysis of cell viability and proliferation of C2C12 on day 1, 4 and 7 under both NX and HX using a two-way ANOVA was performed. To assess the number of MyoD (day1 and day4) and Myogenin (day7) positive C2C12 cells under both experimental conditions, a two-way ANOVA and independent t test, respectively, were carried out. To evaluate the expression of MD (*MyoD* and *Myogenin* at day 7), AD (*PPARγ* and *FABP-4* at day 14) and OD (*OC* and *OP* at day 21) relative markers together with *HIF1α* under both experimental conditions, a two-tailed t test was performed. To examine the effect of combined MD protocol and HX condition on the morphometric parameters of the myotubes including number, length and size, a two-way ANOVA was carried out. Multiple comparisons were tested using Tukey’s and Sidak’s post hoc test. All the data are presented as mean ± SEM and *p* value $\le$ 0.05 was considered to be significant. The analyses were carried out using GraphPad Prism 7.0 (La Jolla, Canada).

## Results

### Effect of combined HX and ectopic differentiation on the myogenic capacities of SC in their microenvironment

**Fig. 1:**
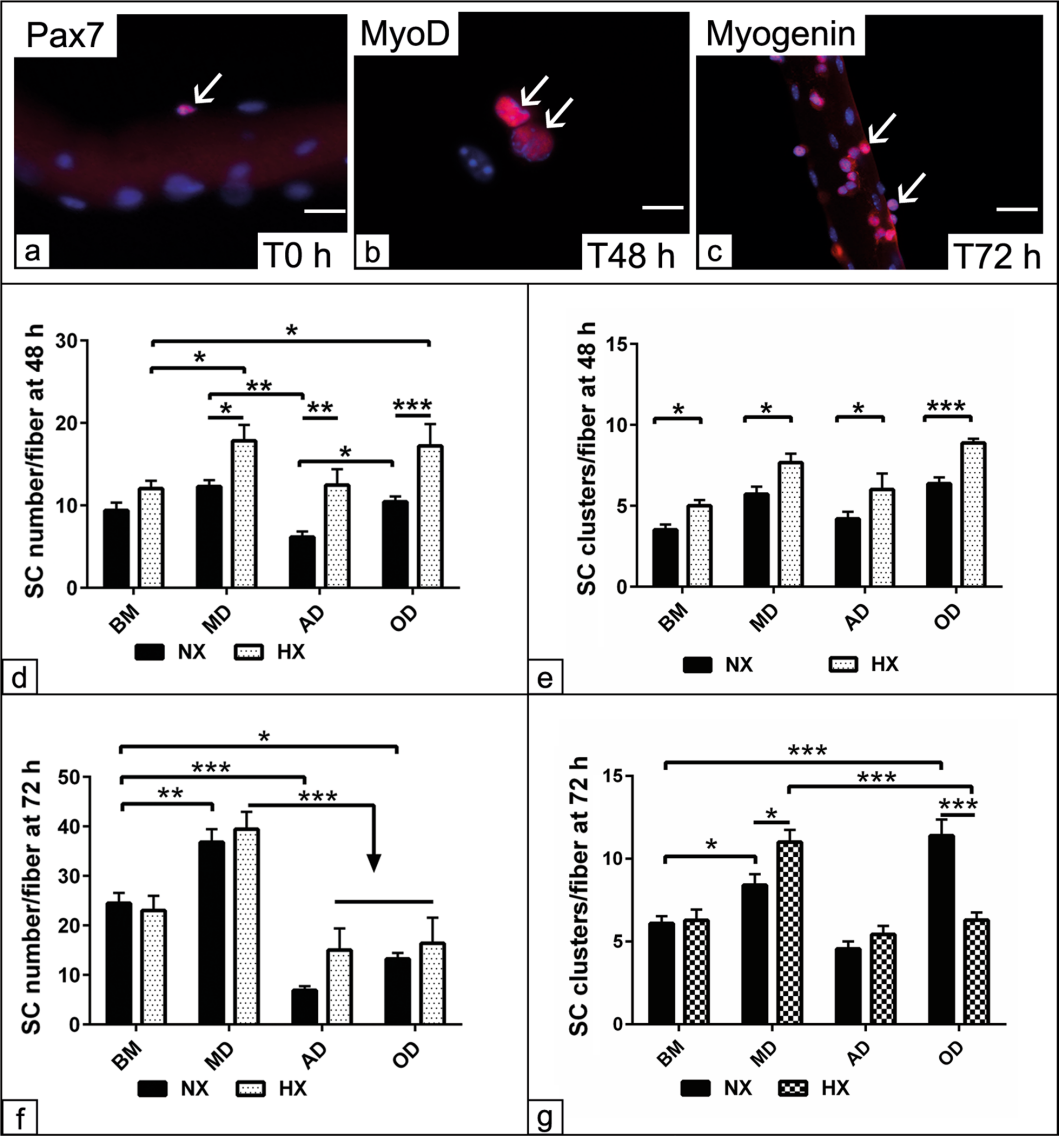
Effect of combined HX and ectopic differentiation on the myogenic capacities of SC in their microenvironment. **a** Immunofluorescence show SC positive for Pax7 at time 0 (T0 h), **b** MyoD at 48 h (T48) and **c** Myogenin at 72 h (T72) in red. SC/fiber were cultivated in basal medium (BM), myogenic differentiation (MD), adipogenic differentiation (AD) and osteogenic differentiation media up to 72 h under both normoxic (NX) and hypoxic (HX) culture conditions. **d** Quantification of the total number of MyoD positive cells per fiber (n = 20 fibers) at 48 h. **e** Quantification of cell clusters positive for MyoD per fiber at 48 h. **f** Quantification of the total number of cells positive for Myogenin per fiber (n = 20 fibers) at 72 h. **g** Quantification of cell clusters positive for Myogenin per fiber at 72 h. All data presented as mean ± SEM. *p < 0.05, **p < 0.01, ***p < 0. 001. DAPI was used as a nuclear counterstain (blue). Scale bar = 10 µM

A multitude of evidence demonstrated SC performance during MD under standard culture condition. However, the oxygen requirement in the cellular microenvironment is far below compared to the ordinary culture condition. Thus, optimizing the oxygen tension in the physiological niche might alter the cellular activity and impact on their regenerative potential. In order to have a more precise data to mimic the *in situ* condition, we have monitored SC multipotency not only under HX but also when the SC remain in contact with their mother fibers. To visualize SC activation, motility, proliferation and differentiation on top of the muscle fiber under culture condition, a live cell imaging up to 72 h was performed (Additional file 1: Video S1). SC identification and tracking was performed using immunohistochemistry for the specific myogenic markers; Pax7, MyoD and Myogenin up to 72 h (Fig. 1a–c). The data showed that HX increased the number of MyoD positive cells per fiber following MD (*p* < 0.05), AD (*p* < 0.01) and OD (*p* < 0.001) condition at 48 h compared to matched cells under NX. By comparing with non-induced cells in BM, HX increased the number of MyoD positive cells adopted for MD and OD (*p* < 0.05) but not those cells undergone AD condition (Fig. 1d). The activated cells migrate along the fiber to form regenerative clusters at the target sites. By counting the number of clusters per fiber at 48 h demonstrated that HX regardless of the induction medium for differentiation was able to enhance cell motility and increase cluster formation along the fiber as observed under BM, MD and AD (*p* < 0.05) compared to NX. The distribution of the cell clusters was even more pronounced in the presence of the OD medium (*p* < 0.001) compared to NX condition (Fig. 1e).

After 72 h, SC tend to differentiate via down regulation of Pax7 and MyoD expression. In contrast, alternatively the expression of Myogenin was enhanced. The data showed that the number of Myogenin positive cells per fiber was higher under MD regardless of the hypoxic effect in comparison with either non-induced cells in BM (*p* < 0.01) or those cells cultivated under AD and OD (*p* < 0.001) conditions. Moreover, under NX, the number of Myogenin positive cells in the presence of AD and OD medium was reduced (*p* < 0.001 and *p* < 0.05) in comparison with non-induced cells in BM. In contrast, the Myogenin positive cells were increased under MD (*p* < 0.001) compared to those cells in BM under NX condition (Fig. 1f). Similarly, the number of SC clusters was increased in the presence of MD under HX (*p* < 0.05) compared to NX condition as the data shown at 48 h. Unexpectedly, the number of SC clusters was increased in the OD medium under NX either in comparison with matched cells under HX (*p* < 0.001) or the non-induced cells in BM (*p* < 0.001) (Fig. 1g). The data revealed enhanced cell proliferation and cluster distribution under HX for all differentiation condition at 48 h that might suggest improved cell proliferation. Furthermore, the reduction in Myogenin positive cells following AD and OD compared to MD under HX at 72 h could be indicative for SC multipotency. The data point out that using OD medium under HX restricts SC cluster formation compared to NX culture.

### Effect of HX on quiescence and terminal differentiation of SC in their native substrate

**Fig. 2:**
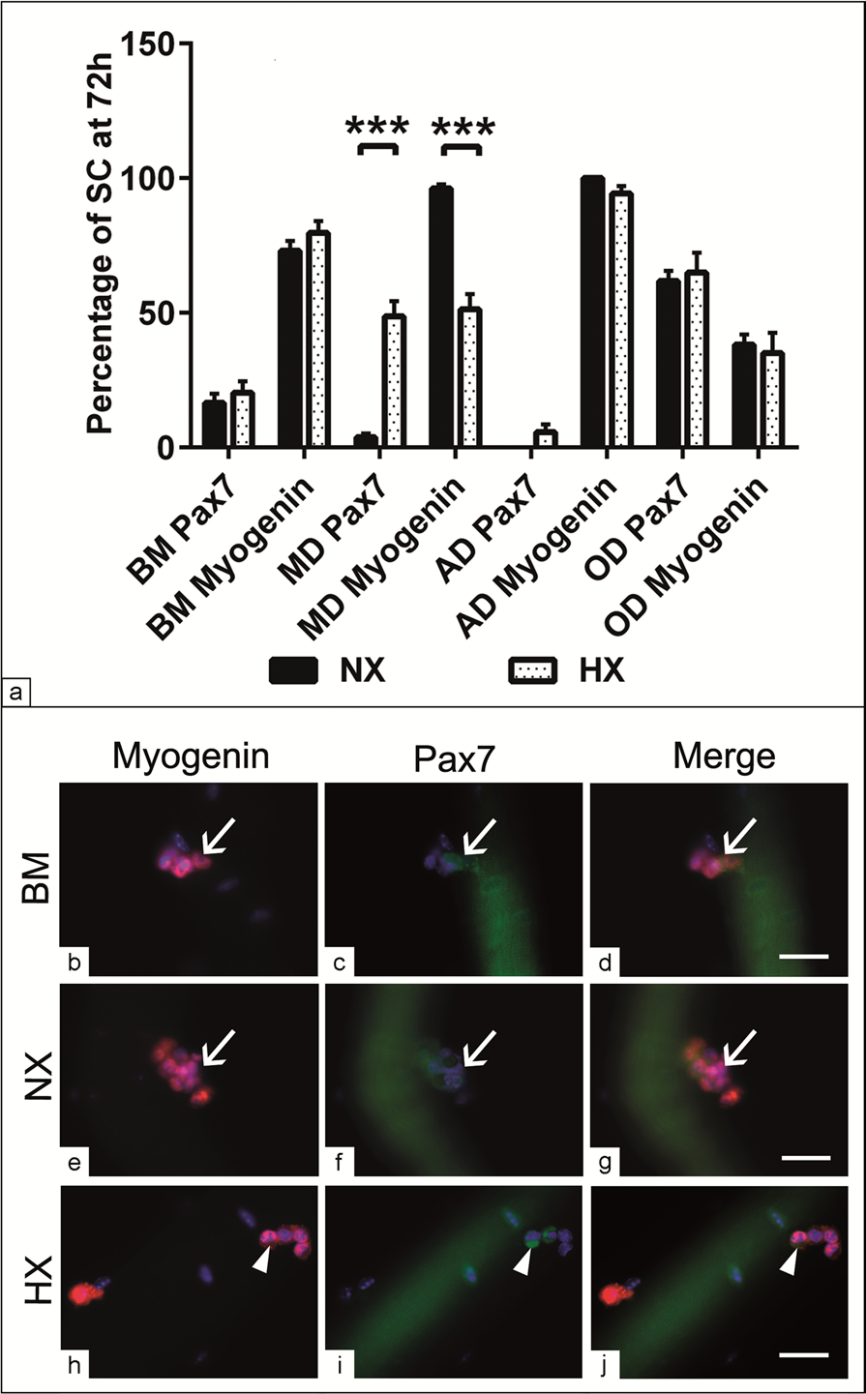
Effect of HX on quiescence and terminal differentiation of SC in their native substrate. **a**–**j** SC/fiber (n = 15–20) were cultivated in BM, MD, AD, OD media in 24-well culture plates in triplicate under both NX and HX conditions for 72 h. **a** Average percentage of Pax7 and Myogenin positive cells per fiber at 72 h. HX reduces the percentage of Myogenin positive cells and increased the percentage of Pax7 positive cells compared to NX condition. **b**–**j** A double immunofluorescence of SC/fiber show Myogenin (red) and Pax7 (green) positive cells (arrow). Note the cells positive for Myogenin (red, arrow head) and Pax7 (green, arrow head) under HX condition at 72 h (**h**–**j**). All data presented as mean ± SEM. *p < 0.05, **p < 0.01, ***p < 0. 001. DAPI was used as a nuclear counterstain (blue). Scale bar = 10 µM

In order to examine whether HX modulate myogenic relative markers in the course of MD, a double immunofluorescence to quantify Pax7 and Myogenin positive cells was carried out at 72 h. The analysis showed that the percentage of cells positive for Myogenin following MD was less under HX (*p* < 0.001) in comparison with matched induction under NX condition. In contrast, the percentage of Pax7 positive cells was higher when MD was carried out under HX (*p* < 0.001) in comparison with NX. Regardless to the influence of the O_2_ concentration, no significance were detected in the percentage of Pax7 and Myogenin positive cells in BM, AD and OD between both experimental conditions (Fig. 2a, b–j). The data point out that HX might interfere with MD via persistent cyclic progression that maintains Pax7 expression.

### HX impairs myogenic differentiation and promotes the multipotency of SC in a monolayer culture

**Fig. 3:**
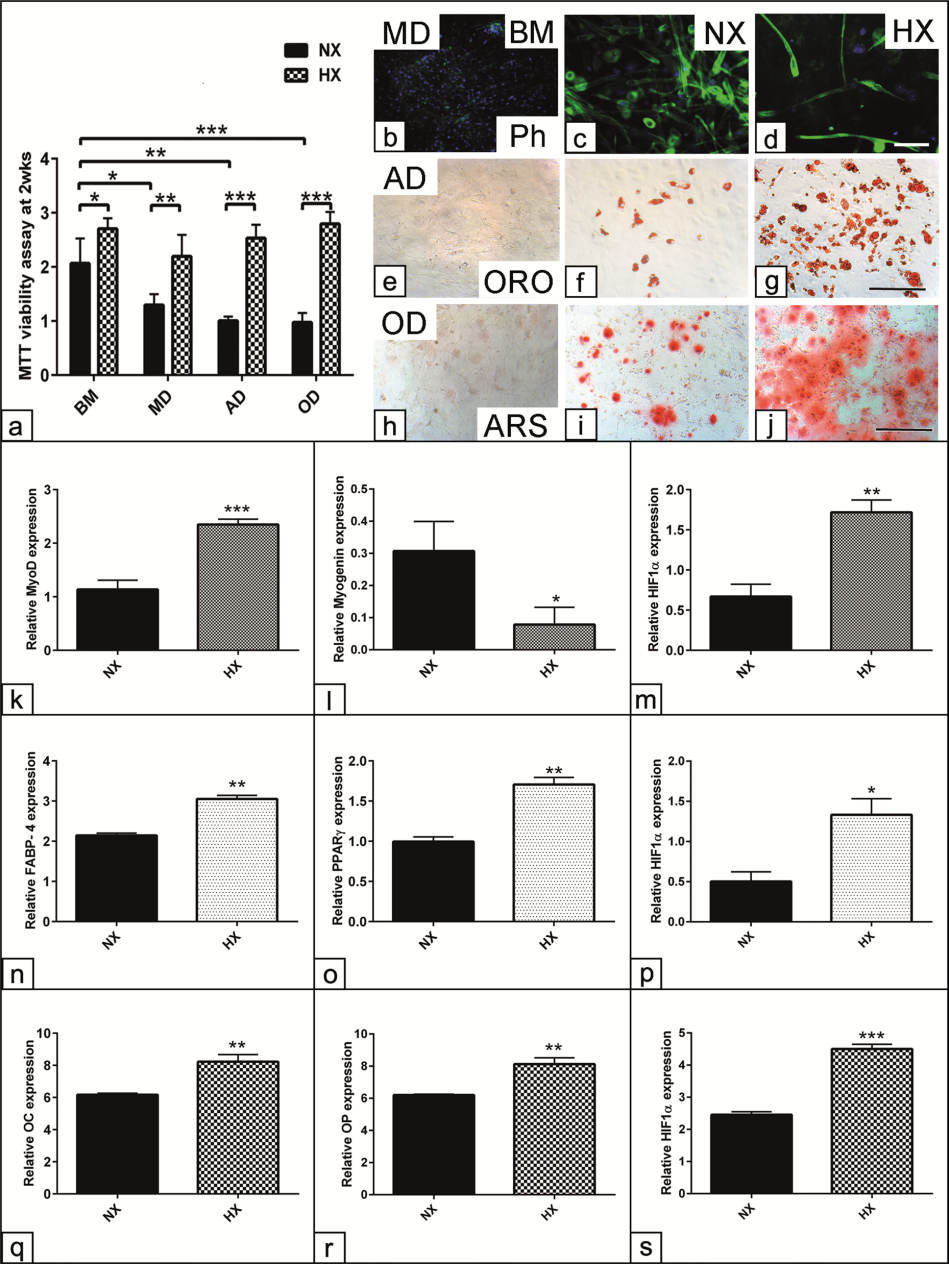
HX impairs myogenic differentiation and promotes the multipotency of SC in a monolayer culture. **a**–**s** Skeletal muscle-derived SC were seeded 1 × 10^4^ cells/well in GM for 48 h and then were allowed to differentiate into myogenic (MD), adipogenic (AD) and (OD) fate using relative induction medium up to 21 days under both NX and HX conditions. **a** Measurement of cell viability under combined differentiation medium and HX culture condition using after two weeks using MTT assay. The absorbance was measured at 570 nm wavelength. **b**–**d** Myotubes formation stained with phalloidin (Ph, green) at day 7 after MD. **e**–**g** Adipocytes contain fat vacuoles stained with Oil Red O (ORO, red) after two weeks of AD. **h**–**j** Matrix mineralization stained with Alizarin Red S (ARS, red) after three weeks of OD. **k**–**s** Quantitative RT-PCR of cells lysates show the relative expression of the MD markers; MyoD and Myogenin (**k**, **l**) at day 7, the AD markers; *FABP-4* and *PPARγ* (**n**, **o**) at day 14 and OD markers; Osteocalcin (*OC*) and Osteopontin (*OP*, **q**, **r**) at day 21 after differentiation induction. **m**, **p**, **s** Quantification of *HIF1α* relative expression in the course of MD, AD and OD induction. Non-induced cells cultivated in parallel in basal medium (BM) were used as negative controls. All data presented as mean ± SEM. *p < 0.05, **p < 0.01, ***p < 0. 001. DAPI was used as a nuclear counterstain (b, c, d, blue).Scale bar in b, c, d = 100 µm and in e–j = 200 µm

To have an overview about the effect of long-term HX exposure on the MD and multipotency of SC, isolated SC were allowed to differentiate as monolayer in MD, AD and OD medium for up to 21 days. Evaluation of cell viability under HX pointed out marked differences in comparison with NX culture condition. The cells undergone MD, AD and OD have shown reduction in cell viability already after two weeks under NX culture (*p* < 0.05, *p* < 0.01 and *p* < 0.001) in comparison with non-induced cells in BM. In contrast, the cells cultivated under HX demonstrated increased cell viability even in parallel with differentiation induction for all experimental groups (*p* < 0.01, *p* < 0.001) as shown for MD, AD and OD in comparison with matched cells under NX (Fig. 3a). Morphological assessment for MD at day 7 using phalloidin staining demonstrated that HX reduces the number of myotubes formation indicative for MD (*p* < 0.05) in comparison with NX condition (Fig. 3b c, d and Additional file 2: Fig. S1a). In contrary, using AD medium, there were numerous adipocytes with larger fat vacuoles under HX in comparison with NX condition as shown for ORO staining at day 14 (Fig. 3e–g). Similarly, OD medium drives a higher matrix mineralization by calcium Ca^2+^ deposition as shown after ARS staining in comparison with NX condition at day 21 (Fig. 3h–j). In order to confirm these histological observations, semi-quantitative analysis of the matrix mineralization and fat vacuoles formation following OD and AD induction, respectively, up to day 21 was performed. The data revealed increased Ca^2+^ deposition following OD at days 14 and 21 under HX (*p* < 0.01 and *p* < 0.001) compared to matched condition at day 7. Moreover, promoted Ca^2+^ deposition under HX at day 21 (*p* < 0.001) compared to the same time point under HX (Additional file 2: Fig. S1b). Similarly, the analysis showed a higher AD capacity for those cells induced under HX at days 14 and 21 (*p* < 0.05 and *p* < 0.001) compared to matched cells under NX condition (Additional file 2: Fig. S1c). The morphometric analysis revealed that under HX, an increase in the number of adipocytes (*p* < 0.001) compared to those cells under NX following AD at day 21 (Additional file 2: Fig. S1d). Moreover, the data revealed increases in the size of the fat vacuoles per adipocyte (*p* < 0.01) but not the number of individual vacuoles per adipocyte under HX condition (Additional file 2: Fig. S1e, f).

At the molecular level, a detailed quantification for the MD, AD and OD relative markers expression using RT-qPCR was carried out. Quantification of the relative mRNA revealed that HX upregulated *MyoD* and *HIF1α* (*p* < 0.001, *p* < 0.01) expression compared to NX condition at day7 of MD (Fig. 3k–m). In contrast, the expression of differentiation marker *Myogenin* was downregulated (*p* < 0.05) at the same condition under HX. On the other hand, the expression of *FABP-4*, *PPARγ* and *HIF1α* were upregulated at day 14 of AD (*p* < 0.01, *p* < 0.05) under HX compared to matched cultivation in NX condition (Fig. 3n–p). Similarly, the expression of osteogenic common markers was evaluated after 21 days under HX. The analysis revealed upregulated *OC*, *OP* and *HIF1α* expression under HX (*p* < 0.01, *p* < 0.001) in comparison with the cells under NX condition (Fig. 3q–s). The data point out that although HX impaired MD alternatively, the cells were more prone to the AD and OD lineages under respective induction.

### Effect of HX on proliferation capacity and myogenic commitment of C2C12

**Fig. 4:**
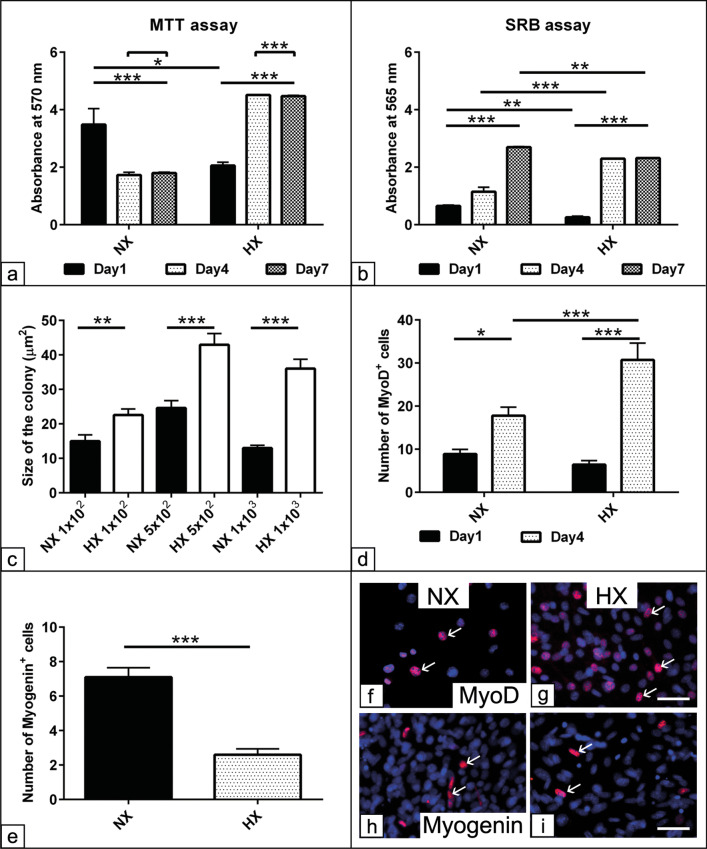
Effect of HX on proliferation capacity and myogenic commitment of C2C12. Mouse myoblasts C2C12 were seeded 1 × 10^4^ cells/well in GM for 1, 4 and 7 days under both NX and HX conditions. **a** MTT assay shows cell viability up to day 7 under HX compared to NX condition. The absorbance was examined at wavelength 570 nm. **b** SRB assay indicative for total protein contents reveals the cell number after days 1, 4 and 7 under HX compared to NX condition. **c** Measurement of C2C12 colony size (µm^2^) at 1 × 10^2^, 5 × 10^2^ and 1 × 10^3^ seeding densities under HX compared to NX condition. C2C12 were seeded at various densities in GM in T-25 culture flasks (n = 3 per experimental group) for 7 days under both NX and HX conditions. The colonies were fixed in 4% PFA for 10 min and then were stained with 5 mg/mL crystal violet in 2% ethanol for 8 min. **d**–**i** Evaluation of the myogenic commitment of C2C12 under HX. C2C12 were seeded 1 × 10^4^ cells/well in triplicates with myogenic differentiation medium up to day 7 under both NX and HX conditions. **d** Number of MyoD positive cells after day-1 and day-4 post-induction. **e** Number of Myogenin positive cells at day-7 post-induction. **f**–**i** Representative immunofluorescence shows MyoD (red) and Myogenin (red) positive cells (arrow) after 3 days under both NX and HX conditions. All data presented as mean ± SEM. *p < 0.05, **p < 0.01, ***p < 0. 001. DAPI was used as a nuclear counterstain (blue). Scale bar = 20 µM

To have a precise overview regarding the effect of HX on MD, C2C12 myoblasts cell line was deployed to examine cell viability and proliferation capacities as well as the efficiency of MD protocols. Analysis of cells viability up to day 7 revealed a reduced cell viability after day 1 under HX (*p* < 0.05) followed by a compensatory increases in cell viability at day 4 and day 7 either in comparison with the cells under NX (*p* < 0.001) or after day 1 of HX (*p* < 0.001). The analysis showed a significant interaction between the effect of HX modulation and the time scale on the cell viability (*p* < 0.001) that point for alteration in the cell performance under HX (Fig. 4a). To evaluate whether enhanced cell viability under HX is due to improved metabolic activity without a real increases in the cell number or there is an increased cell proliferation. Although a reduction in the total protein contents indicative for reduction in the cell number after 1 day under HX (*p* < 0.01), an increase after day 4 (*p* < 0.001) could be detected in comparison with cells under NX. In contrast, cells under HX showed a lower cell number (*p* < 0.01) when compared to day 7 under NX. The analysis revealed a significant interaction (*p* < 0.001) between the effect of HX and the experimental time scale on the cell number (Fig. 4b). The data point for that although cells under HX showed promoted cell viability at day 7, the cell number was even less which might suggest superior effect of HX on metabolic activity. Along the line, C2C12 myoblasts were evaluated under HX using a colony formation assay. The analysis revealed a stable and progressive increase in the colony size indicative for cell proliferation under HX (*p* < 0.01, *p* < 0.001 and *p* < 0.001) at 1 × 10^2^, 5 × 10^2^ and 1 × 10^3^ seeding densities compared to NX culture condition. A double increases in the colony size at 5 × 10^2^ and 1 × 10^3^ seeding densities was detected under HX compared to cells under NX (Fig. 4c).

To examine the cell positivity for myogenic markers, Pax7, MyoD and Myogenin under HX, an immunofluorescence staining was conducted. The results showed no Pax7 positive cells under both experimental conditions up to day 4; however, MyoD and Myogenin positive cells were detected in both conditions (Fig. 4f–i). The analysis revealed increases in the number of MyoD positive cells at day 4 (*p* < 0.05, *p* < 0.001) compared to day 1 in both NX and HX conditions, respectively. Furthermore, the number of MyoD positive cells was higher under HX at day 4 (*p* < 0.001) in comparison with NX at the same time point (Fig. 4d). In contrast, the number of Myogenin positive cells were significantly higher under NX at day 7 (*p* < 0.001) compared to HX (Fig. 4e).

### Evaluation of various protocols on MD capacity of C2C12 cells under HX condition

**Fig. 5:**
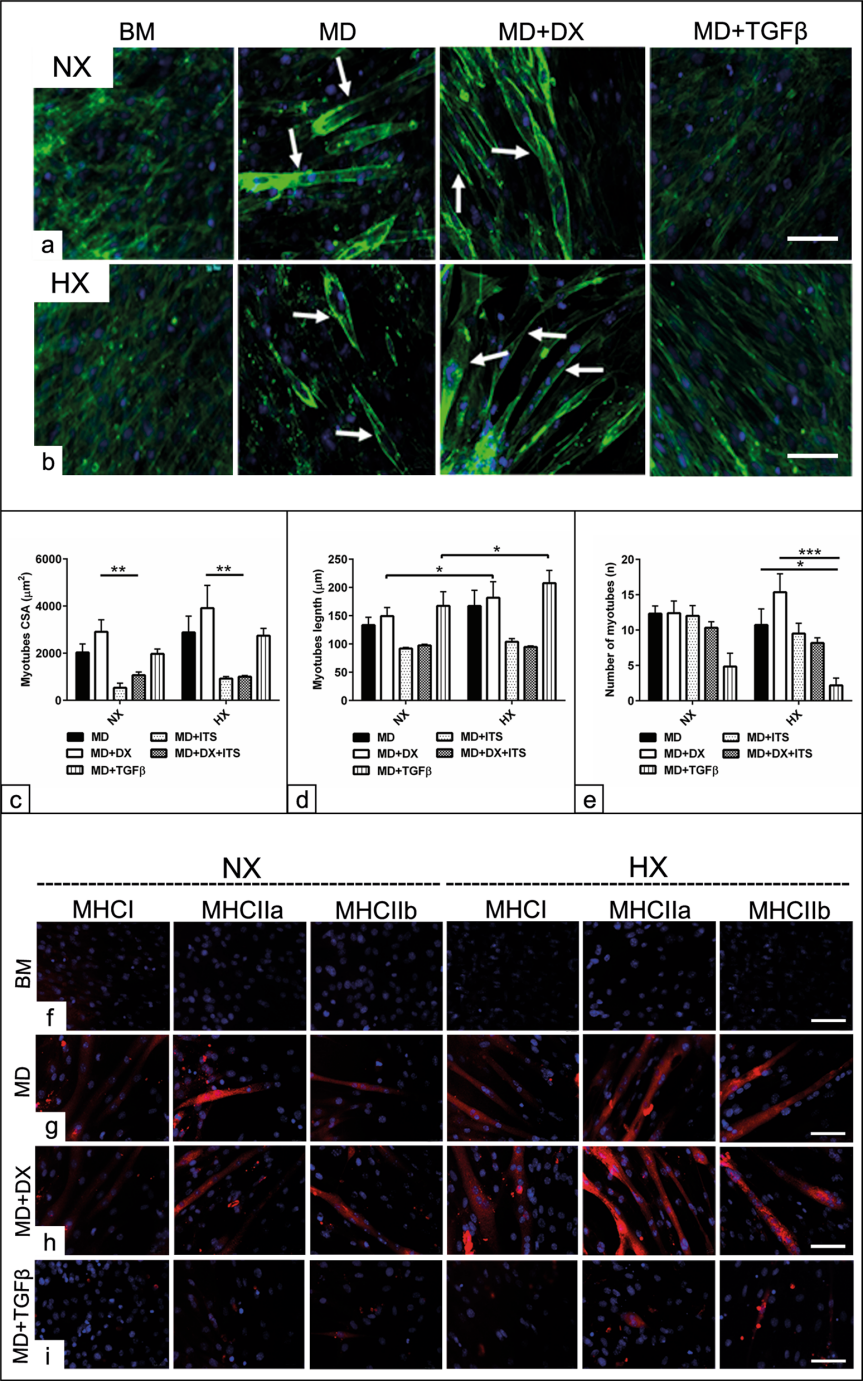
Evaluation of various protocols on MD capacity of C2C12 cells under HX condition. C2C12 were seeded 1 × 10^4^ cells/well in GM for 48 h then were differentiated into myogenic fate using only standard myogenic differentiation (MD) medium or using combined MD medium together with dexamethasone (MD + DX), MD + TGFβ, MD + ITS and MD + DX + ITS for 14 days under both NX and HX conditions. **a**, **b** C2C12 cells stained with phalloidin actin filaments stain (green) show myotubes formation indicative for myogenic differentiation after day 7 under NX and HX conditions. Combined MD + DX based protocol enhances myogenic differentiation in comparison with either MD or MD + TGFβ. Non-induced cells cultivated in parallel in basal medium (BM) were used as negative controls. **c**–**e** Morphometric analysis reveals the size (µm^2^, c), length (µm, d) and number (n, e) of the myotubes per microscopic field (n = 10) following myogenic differentiation for 7 days using MD, MD + DX, MD + TGFβ, MD + ITS and MD + DX + ITS-based protocols under both NX and HX conditions. **f**–**i** Immunofluorescence of C2C12 cells show MHCI, MHCIIa, and MHCIIb positive myotubes (red) after differentiated in MD (**g**), MD + DX (**h**) and MD + TGFβ (**i**)-based protocols under both NX and HX conditions. Non-induced cells cultivated in parallel in basal medium (BM) were used as negative controls (**f**). All data presented as mean ± SEM. *p < 0.05, **p < 0.01, ***p < 0. 001. DAPI was used as a nuclear counterstain (blue). Scale bar = 20 µM

In order to assess whether HX modulate the efficiency of the MD, various protocols were carried out. Phalloidin staining demonstrated less myotube formation under HX in the presence of standard MD medium; however, combined MD medium with dexamethasone (DX) was able to increase myotube formation (Fig. 5a, b). Morphometric analysis revealed no detectable alterations in the number of myotubes for the selected protocols under NX. In contrast, a significant reduction in myotube number was observed in the presence of MD + TGFβ (*p* < 0.05) in comparison with either MD medium or MD + DX under HX conditions. Although myotube number increased using the protocol of combined MD + DX compared to MD alone, was not statistically significant (Fig. 5e). The data showed increased myotubes length (µm) in the MD + DX and MD + TGFβ-based protocols under HX (*p* < 0.05) in comparison with the same protocol under NX (Fig. 5d). In order to examine whether selected protocols alter the size of the formed myotubes, the area of the individual myotubes (µm^2^) was measured. The analysis revealed significant increases in the myotube size under HX (*p* < 0.05) when MD medium was combined with DX in comparison with either MD + ITS or MD + DX + ITS-based protocols. By combining MD medium with TGFβ, no change was detected in the size of the myotube in both experimental conditions (Fig. 5c). In order to examine the presence of the contractile proteins under HX, immunofluorescence for the common MHC in the muscles was carried out. The morphologic observation demonstrated that after 14 days under NX, few myotubes were positive for MHCI, IIa and IIb either in the presence of MD or MD + DX- based protocols compared to non-induced cells in BM. In contrast, the same protocols under HX showed marked increases in the MHCI, IIa and IIb positive myotubes in comparison with both non-induced cells in BM and as well as the cells maintained under NX condition (Fig. 5f, g). No morphological difference under both O_2_ conditions when TGFβ was added to the MD medium (Fig. 5i).

## Discussion

Cell-based therapy has received much attention in the last few decades. Optimizing the isolation, characterization and expansion procedures would increase the efficiency of stem cell for clinical applications in musculoskeletal disorders [42, 43]. It is worth mentioning that the impaired cell viability, low survival rate and improper microenvironment significantly reduce the efficiency of stem cells for regeneration. Consequently, basic research plays a pivotal role to optimize cellular microenvironment to mimic the physiological niche. We hypothesized that low oxygen concentration to a more physiological levels would improve regeneration capacities of SC.

Our data reveal an enhanced cell viability, distribution and proliferation under HX when SC remained in contact with their own muscle fiber. The mechanism involved in promoted cell proliferation is more likely due to low O_2_ tension activates cell cycle-dependent molecules leading to enhanced cell proliferation and motility along the muscle fiber. In agreement with our thought, it has been reported that isolated myoblasts from aged rat showed increased cell proliferation and larger myotube under HX in comparison with the standard culture condition [44]. A similar study has shown that neural stem cells have a higher proliferation and survival rate under HX [45, 46]. We believe that HX causes intracellular molecular adaptations to overcome lack of O_2_ tension. In the same line, it was found that the level of G1/S cyclins and cyclin-dependent kinases was increased and the level of cell cycle inhibitor p27 was decreased together with elevated Akt phosphorylation under HX up to 72 h [28]. This is in consistent with the downregulation of *Myogenin* under HX as shown in our data suggesting that HX might interfere with the cell withdrawal from the cyclic proliferation. An important report revealed that HX inhibits the expression of the MD molecules via blocking the expression of cell cycle-dependent kinase inhibitor p21 and p27 in addition, it prevents the product of the *retinoblastoma* gene leading to a failure of cell cycle withdrawal and terminal differentiation. Furthermore, HX promotes MyoD degradation which prevents the recruitment of the differentiation marker [33]. In agreement with this hypothesis, it has been documented that under 0.5–2% O_2_ level, *MyoD* expression was transiently blocked causing a delayed *Myogenin* expression in C2C12 myoblasts, the author concluded that HX induction initiates a transient acetylation of the *MyoD* promotor [47].

The data revealed that the number of Myogenin positive cells was decreased to the control level when AD and OD medium was used that might indicate inhibition of MD alternatively, induction of AD and OD fates. Surprisingly, the number of clusters per fiber in the presence of OD was higher under NX compared to HX. However, the possible explanation that cell viability criteria including activation and motility along the fibers was suppressed under combined HX exposure together with OD induction elements. The other possibility that under combined HX and OD medium, the cells showed a robust activation of the OD that might restrict the cell clustering along the fibers and impair their motility.

The results revealed an increased number of Pax7 positive cells after 72 h under HX. These data could point out that HX promotes SC proliferation rather than terminates the myogenic differentiation. In this respect, it was found that HX enhances the stemness properties that facilitates the in vitro reproducibility of muscle progenitors before further therapeutic applications [44]. Under HX, SC undergo symmetric division that facilitates self-renewal and quiescence rather than an asymmetric division toward MD. Such mechanism is modulated by *Notch* signaling via inhibiting miR-1 and miR-206 activities through canonical Hes/Hey proteins causing increases in *Pax7* expression [48]. Similarly, by using a myogenic cell lineage overexpressing *Notch*, it was found that *Pax7* was directly regulated via *Notch* expression that leads to promoted self-renewal of SC [49]. Along this line of argumentation, using human SC for tissue engineering constructs has revealed that HX condition improves the myogenic proliferation capacity via upregulations of *Pax7*, *Myf5* and *MyoD* expression, while it maintains the quiescence properties of the SC population [50]. It is worth mention that HX recruits myoblasts transition from differentiation to proliferation by decreasing p38 phosphorylation in the differentiating cells [51]. Our data suggest that the cells lose Pax7 at later passage in C2C12 (P8-9) additionally used in the present study. In the same line, it has been reported although quiescent cells and proliferating myoblasts express Pax7, proliferating cells downregulate Pax7 expression after a certain round of multiplication [52].

Cultivation of the C2C12 myoblasts under HX revealed a transient reduction after 24 h followed by promoted cell viability up to day 4. The initial reduction in the cell viability under HX could be an adaptational mechanism to compensate the lack of oxygen. This finding is partially in accordance with a previous report indicating that the proliferation of humans myoblasts was slightly lower under 5% O_2_ HX, whereas the cell proliferation become higher under HX at later time points compared to NX [53]. Similar studies have revealed that HX enhances the proliferation of mouse primary isolated myoblasts [30], bovine myoblasts [29], rat primary isolated myoblasts [31] and human myoblasts [53]. These data suggest that HX might enhance the metabolic activity of stem cells leading to a higher viability even without a comparable increase in cell number as shown at day 7. This phenomenon could be a crucial prerequisite to improve the regenerative ability of stem cell.

The enhanced proliferative capacity of C2C12 under HX in terms of a stable and progressive increases in the colony size indicating the positive effect on cell viability at various seeding densities. These data suggest that HX enhanced the proliferation ability of the individual cells to form larger colonies compared to NX as previously reported in rat [44] and in human myoblasts [27]. We believe that quiescent SC require low O_2_ level for their maintenance in an in vivo microenvironment, thus the effect of HX would support their quiescence. Along the line, a study concluded that SC express *Hypoxia inducible factor 2A* (*HIF2A*) which promotes their quiescence and prevent the MD via a mechanism including *Spry 1* gene activation by *HIF2A*. The inhibition of *HIF2A* improves the regenerative capacity via enhanced cell proliferation and differentiation which might have a therapeutic value to promote muscle regeneration [54]. In the present study, a 3% O_2_ level was sufficient to observe an enhanced cell viability in C2C12 cells; however, below this, threshold might have a toxic or damaging effect to the cells. It was found that severe HX (1% O_2_) level impaired myoblast function due to aberrant epigenetic modulation of the autophagy including dephosphorylation of *Glycogen synthase kinase 3β* (*GSK3β*) and inactivation of *Notch* signaling [55].

Multipotential induction under HX revealed a reduction in myogenic differentiation capacity. In agreement with our results, it was reported that myotube formation by C2C12 myoblasts cultured in differentiation medium for 3 days was lower under HX compared to NX conditions [29]. Similarly, neither myotube formation nor MHC expression could be detected in C2C12 cells and in the L6E9 rat myoblast cultured in a differentiation medium for 1 and 2 days under HX [33]. Our data are in agreement with previous report explaining the suppressive effect of HX on MD. It was found that HX upregulates *histone deacetylases* (*HDACs*) causing impaired myogenesis [55]. In the same line, lack of O_2_ inhibits protein synthesis as well as signaling including the phosphatidylinositol 3-kinase (PI3K)/AKT/mTOR pathway that plays a role to maintain MyoD1 stability and translation via mammalian target of rapamycin1 (mTOR1) activation [51]. Additionally, the modulated PI3K/AKT signaling was due to sensitivity of insulin-like growth factor receptor (IGFIR) to O_2_ deprivation [56].

For technical reason, there was a limited capacity to cultivate SC/ fiber for more than 72 h. Alternatively, long-term HX condition has been applied on isolated SC as monolayer culture to monitor AD and OD events. The data point out that SC are more liable for AD under HX as the data revealed increases in the number of adipocytes and the size of fat vacuoles per adipocytes. Similarly, the enhanced OD under HX as shown by semi-quantitative analysis of ARS staining. These data could be an adaptive mechanism of SC to compensate a lack of oxygen tension. A large body of evidence suggest that O_2_ concentration modulates the cell differentiation fate including adipogenesis, chondrogenesis and neural differentiation [46, 57, 58]. Moreover, a study has revealed the hypoxic nature of the local microenvironment within the adipose tissue in obese mice supporting our data [59]. Interestingly, it was found that HX induction promotes osteogenic differentiation via *HIF-1*-dependent mechanism in bone marrow-derived mesenchymal stem cells (MSCs) in humans suggesting their potential benefit for bone regeneration [60]. As our data shown, *HIF1α* was upregulated in the course of AD and OD under HX condition which suggest that *HIF-1α* mediates SC multipotency via modulation of energy metabolism and perhaps angiogenesis to counteract the effect of local ischemia. In the same line, a recent study concluded that upregulation of *HIF2* expression in the adipocytes drives adipogensis in white fat in contrast, lack of its expression in brown fat enhances glucose tolerance in vivo in mice [61]. In the present study, the cells were exposed to continuous HX up to 21 days. A similar study investigating the effect of cyclic HX on OD in mesenchymal stem cells revealed an improvement of bone condition with ageing due to the inhibition of bone resorption and enhanced mineral density [62]. In the same line, similar study comparing the adipogenic and osteogenic capacities of both compact bone and bone marrow MSCs reported that although both cell populations revealed similar adipogenic ability, compact bone-derived MSCs showed a superior osteogenic capacity under HX [63]. Furthermore, it has been reported that HX enhances *vascular endothelial growth factor* (*VEGF*) and *interleukin 6* (*IL6*) expression as well as it coordinates stem cells homing and mobilization [64]. Along the line, using biomaterials scaffold incorporated with HX inducing agents (Desferoxamine) to enhance OD of adipose tissue-derived mesenchymal stem cells reported the efficiency of scaffolds to upregulate *HIF1α* and *VEGF* expression and to improve OD in humans [65].

The data revealed a positive effect of supplementing the MD medium with dexamethasone under HX on the MD. Recent study concluded that dexamethasone enhances muscle regeneration in mice via improving the kinesin-1 motor activity that plays a central role to stimulate myogenic contractile elements expression, myoblasts fusion and formation of polarized myotubes [66]. The time of dexamethasone treatment either before myogenic induction or after the onset of myotubes might be a critical approach for the outcome. It was found that treatment with dexamethasone after differentiation produced a thinner myotube as well as decreased MHC proteins. In contrast, treatment with dexamethasone before the differentiation not only increased the number and size of myotubes, but also the level of MHC proteins [67]. Myogenesis can adapt to hypoxic conditions; a report revealed that although MD of C2C12 myoblasts was repressed after day 3 under 0.5% O_2_, an extensive MD was detected up to day 12 [47]. The present data revealed that the enhanced MD with dexamethasone was blocked by adding ITS suggesting a suppressive effect on MD. A report revealed that glucocorticoids showed anti-adipogenic and pro-myogenic activities that promote the terminal myogenic differentiation; such effect was glucocorticoids receptor-dependent [68]. In contrast, combined MD together with TGFβ inhibits MD under HX. In agreement with our data, it has been found that myoblasts releases Myostatin, a member of TGFβ superfamily under HX which interrupts the negative feedback effect leads to inhibition of cell proliferation and enhances muscle growth [69]. Furthermore, the overexpression of Myostatin reduces the expression of MyoD1 [70] via AKT signaling pathway inhibition [71].

## Conclusion

The present study elucidated the effect of HX on MD of SC. The data revealed that under HX an enhanced cell proliferation, cell motility along the fiber and promoted SC quiescence as well as an impairment of MD. The increased multipotency of SC under HX as shown by enhanced adipogenesis and osteogenesis suggests that targeting HX could be beneficial to improve bone regeneration. The negative effect of HX on the MD was improved by adding dexamethasone to the MD medium. Altogether, the data provide a closer look to the regenerative and multipotential abilities of the myogenic precursors using two culture model to mimic the in vivo and in vitro regeneration under HX. We provide evidence that 3% HX exposure might be beneficial to improve stem cell reproducibility before transplantation also considering HX induction to promote SC multipotency.

## Supplementary Information

**Additional file 1: Video S1**. Monitoring SC on top of the muscle fiber under standard culture condition. Live cell imaging shows SC activation, motility, proliferation and differentiation on top of the muscle fiber under culture condition.

**Additional file 2: Fig. 1**. Quantification of multipotential induction of SC under HX condition. SC were seeded 1 × 104 cells/well (n = 6 per experimental condition) in GM for 48 h then, were kept in myogenic differentiation (MD) medium contained 4.5 g/L glucose DMEM supplemented with 2% horse serum, 2.5 ng/mL human Fibroblast Growth Factor, 1% Sodium pyruvate up to 14 days; adipogenic differentiation medium (AD) composed of 4.5 mg/mL glucose DMEM supplemented with 5% FCS, 1 µM dexamethasone, 5 µg/mL Insulin-transferrin-selenium and 5 µM Rosiglitazone and osteogenic differentiation medium (OD) contained 1mg/mL glucose DMEM supplemented with 5% FCS, 0.1 µM dexamethasone, 250 µM ascorbic acid, and 10 mM β-glycerophosphate up to 21 days under both NX and HX conditions. Non-induced cells kept in basal medium (BM) containing 5% FCS in DMEM were considered controls (a) Number of myotubes per microscopic field (n = 5) show reduced myotubes formation under HX. (b) Semi-quantification of Alizarin Red S staining shows increased Ca+2 deposition under HX indicative for OD after 14 and 21 days. (c) Semi-quantification of Oil Red O staining shows enhanced AD after 14 and 21 days. (d) Number of adipocytes per microscopic field (n = 5) reveals increases of AD under HX. (e) Number of fat vacuoles per adipocyte shows no changes under HX. (f) Measurement of fat vacuoles per adipocyte shows increases of the individual vacuoles size (µm2) under HX. All data presented as mean ± SEM. *= p < 0.05, **= p < 0.01, ***= p < 0. 001.

## Data Availability

The data collected and the analysis performed to generate the manuscript results are available from the corresponding author on reasonable request.

## Abbreviations

SC: Skeletal muscle-derived stem cells
C2C12: Mouse myoblasts cell line
NX: Normoxic condition
HX: Hypoxic condition
MHC: Myosin heavy chain
MD: Myogenic differentiation
AD: Adipogenic differentiation
OD: Osteogenic differentiation
BM: Basal medium
GM: Growth medium
RT: Room temperature
DX: Dexamethasone
Pax7: Paired Box 7
MRF4: Myogenic regulatory factor 4
TGFβ: Transforming growth factor beta
ITS: Insulin–transferrin–sodium selenite
*PPARγ*: *peroxisome proliferator activated receptor gamma*
*FABP4*: *fatty acid-binding protein 4*
*OC*: *Osteocalcin*
*OP*: *osteopontin*
*HIF1α*: *Hypoxia inducible factor 1 alpha*
*HIF2A*: *Hypoxia inducible factor 2A*
*GSK3β*: *Glycogen synthase kinase 3β*
*HDACs*: *Histone deacetylases*
mTOR1: Mammalian target of rapamycin 1
IGFIR: Insulin-like growth factor receptor
*VEGF*: *Vascular endothelial growth factor*
*IL6*: *Interleukin 6*
DAPI: 4,6-Diamidino-2-phenylindole dihydrochloride
SRB: Sulforhodamine B
MTT: (3-(4, 5-Dimethylthiazol-2-yl)-2, 5-Dimethyltetrazolium Bromide) cell viability assay
PFA: Paraformaldehyde
