# Supplementary material for: The effect of hypoxia on myogenic differentiation and multipotency of the skeletal muscle-derived stem cells in mice
Source: Stem Cell Res Ther. 2022 Feb 5;13:56. doi: 10.1186/s13287-022-02730-5 (PMC8817503; doi:10.1186/s13287-022-02730-5)
Supplement: Supplementary file 1 — Additional file 1: Video S1. Monitoring SC on top of the muscle fiber under standard culture condition. Live cell imaging shows SC activation, motility, proliferation and differentiation on top of the muscle fiber under culture condition. [file 13287_2022_2730_MOESM1_ESM.pptx]

## Slide 1
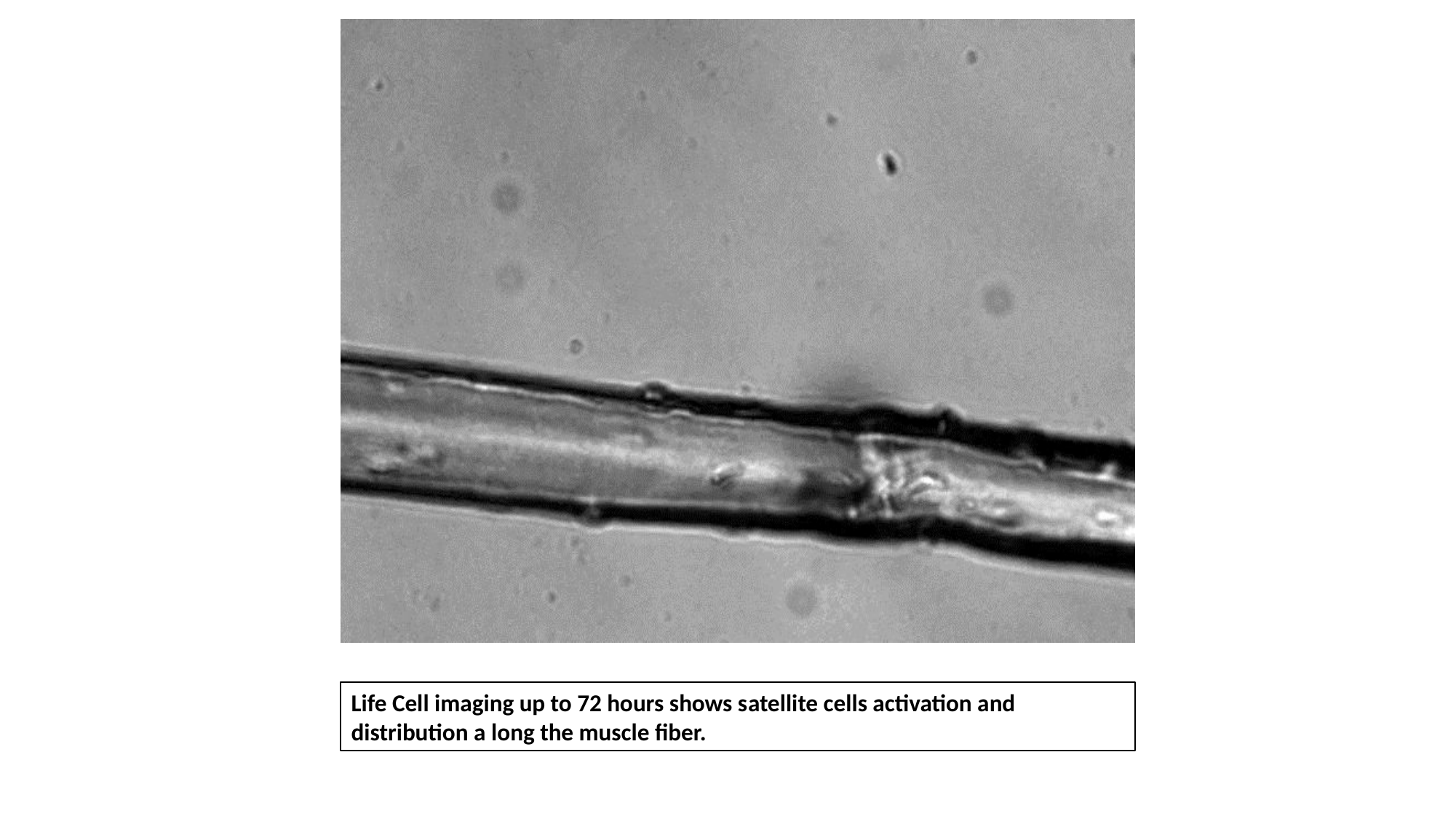

Life Cell imaging up to 72 hours shows satellite cells activation and distribution a long the muscle fiber.
