# Supplementary figures and images for: The effect of hypoxia on myogenic differentiation and multipotency of the skeletal muscle-derived stem cells in mice
Source: Stem Cell Res Ther. 2022 Feb 5;13:56. doi: 10.1186/s13287-022-02730-5 (PMC8817503; doi:10.1186/s13287-022-02730-5)

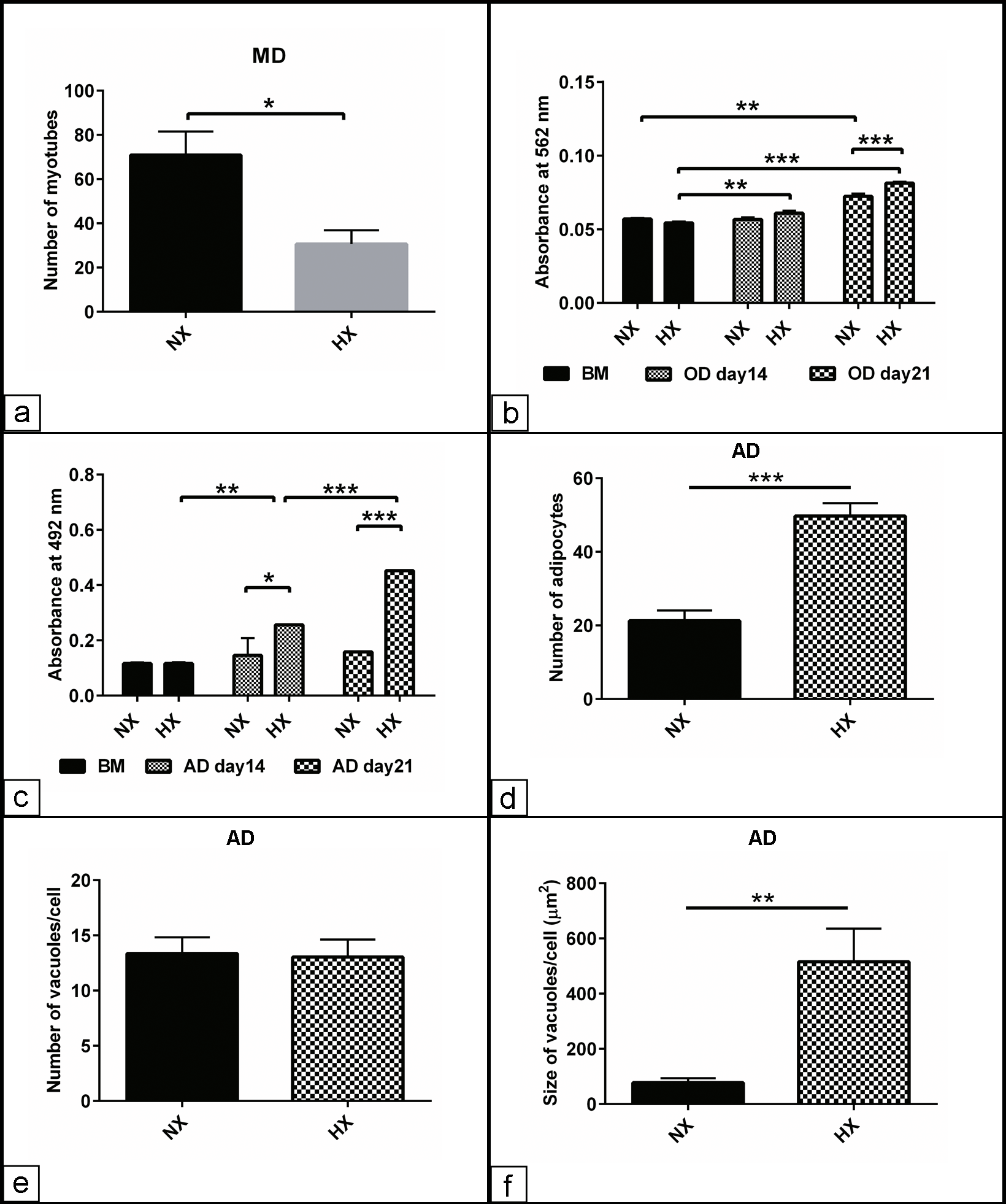

Supplement: Supplementary file 2 — Additional file 2: Fig. 1. Quantification of multipotential induction of SC under HX condition. SC were seeded 1 × 10^4^ cells/well (n = 6 per experimental condition) in GM for 48 h then, were kept in myogenic differentiation (MD) medium contained 4.5 g/L glucose DMEM supplemented with 2% horse serum, 2.5 ng/mL human Fibroblast Growth Factor, 1% Sodium pyruvate up to 14 days; adipogenic differentiation medium (AD) composed of 4.5 mg/mL glucose DMEM supplemented with 5% FCS, 1 µM dexamethasone, 5 µg/mL Insulin-transferrin-selenium and 5 µM Rosiglitazone and osteogenic differentiation medium (OD) contained 1mg/mL glucose DMEM supplemented with 5% FCS, 0.1 µM dexamethasone, 250 µM ascorbic acid, and 10 mM β-glycerophosphate up to 21 days under both NX and HX conditions. Non-induced cells kept in basal medium (BM) containing 5% FCS in DMEM were considered controls (a) Number of myotubes per microscopic field (n = 5) show reduced myotubes formation under HX. (b) Semi-quantification of Alizarin Red S staining shows increased Ca+2 deposition under HX indicative for OD after 14 and 21 days. (c) Semi-quantification of Oil Red O staining shows enhanced AD after 14 and 21 days. (d) Number of adipocytes per microscopic field (n = 5) reveals increases of AD under HX. (e) Number of fat vacuoles per adipocyte shows no changes under HX. (f) Measurement of fat vacuoles per adipocyte shows increases of the individual vacuoles size (µm2) under HX. All data presented as mean ± SEM. *= p < 0.05, **= p < 0.01, ***= p < 0. 001. [file 13287_2022_2730_MOESM2_ESM.tif]
